# The homodimer interfaces of costimulatory receptors B7 and CD28 control their engagement and pro-inflammatory signaling

**DOI:** 10.1186/s12929-023-00941-3

**Published:** 2023-06-28

**Authors:** Andrey Popugailo, Ziv Rotfogel, Michal Levy, Orli Turgeman, Dalia Hillman, Revital Levy, Gila Arad, Tomer Shpilka, Raymond Kaempfer

**Affiliations:** Department of Biochemistry and Molecular Biology, Institute of Medical Research Israel-Canada, The Hebrew University-Hadassah Medical School, 9112102 Jerusalem, Israel

**Keywords:** Inflammatory cytokine storm, Pro-inflammatory signaling, Costimulatory receptors B7 and CD28, Control of B7/CD28 receptor engagement, Receptor homodimer interface mimetic peptides, Regulation of B7/CD28 signaling

## Abstract

**Background:**

The inflammatory response is indispensable for protective immunity, yet microbial pathogens often trigger an excessive response, ‘cytokine storm’, harmful to the host. Full T-cell activation requires interaction of costimulatory receptors B7-1(CD80) and B7-2(CD86) expressed on antigen-presenting cells with CD28 expressed on the T cells. We created short peptide mimetics of the homodimer interfaces of the B7 and CD28 receptors and examined their ability to attenuate B7/CD28 coligand engagement and signaling through CD28 for inflammatory cytokine induction in human immune cells, and to protect from lethal toxic shock in vivo.

**Methods:**

Short B7 and CD28 receptor dimer interface mimetic peptides were synthesized and tested for their ability to attenuate the inflammatory cytokine response of human peripheral blood mononuclear cells, as well as for their ability to attenuate B7/CD28 intercellular receptor engagement. Mice were used to test the ability of such peptides to protect from lethal superantigen toxin challenge when administered in molar doses far below the toxin dose.

**Results:**

B7 and CD28 homodimer interfaces are remote from the coligand binding sites, yet our finding is that by binding back into the receptor dimer interfaces, short dimer interface mimetic peptides inhibit intercellular B7-2/CD28 as well as the tighter B7-1/CD28 engagement, attenuating thereby pro-inflammatory signaling. B7 mimetic peptides exhibit tight selectivity for the cognate receptor in inhibiting intercellular receptor engagement with CD28, yet each diminishes signaling through CD28. In a prominent example of inflammatory cytokine storm, by attenuating formation of the B7/CD28 costimulatory axis, B7-1 and CD28 dimer interface mimetic peptides protect mice from lethal toxic shock induced by a bacterial superantigen even when administered in doses far submolar to the superantigen.

**Conclusions:**

Our results reveal that the B7 and CD28 homodimer interfaces each control B7/CD28 costimulatory receptor engagement and highlight the protective potential against cytokine storm of attenuating, yet not ablating, pro-inflammatory signaling via these receptor domains.

**Supplementary Information:**

The online version contains supplementary material available at 10.1186/s12929-023-00941-3.

## Background

The inflammatory cytokine response is indispensable for protective immunity, yet bacterial and viral pathogens often elicit an exaggerated reaction, ‘cytokine storm’, harmful to the host. Despite recent advances in our understanding of inflammatory signaling, how to prevent a cytokine storm remains a challenge. Here, we focus on how the host inflammatory response is controlled.

Effective T cell activation, resulting in induction of inflammatory cytokines, requires engagement of costimulatory receptor CD28 on the T cell with its B7-1 (CD80) and B7-2 (CD86) coligands on antigen-presenting cells [1–4]. Expressed constitutively, CD28 is a homodimer that enhances the activation of innate and adaptive immune responses [2, 4]. Whereas expression of B7-1 is induced only in the course of an immune response in dependence on CD28 signaling, B7-2 is expressed constitutively [3]; hence, the B7-2/CD28 interaction regulates immediate inflammatory responses [5, 6]. Expression of both B7-1 and B7-2 increases in response to infectious stimuli [3].

In the extracellular domains of CD28, B7-1 and B7-2, the receptor homodimer interfaces are located remotely from the binding sites for their co-ligands [7–10]. Whereas CD28 and B7-1 are covalent homodimers [7, 8], B7-2 forms a weak, noncovalent homodimer and exists mostly as a monomer on the cell surface [6, 11]. Here we demonstrate an essential role for the CD28, B7-1 and B7-2 homodimer interfaces in regulating B7/CD28 receptor engagement and signaling through this costimulatory axis. Our finding is that formation of the B7/CD28 costimulatory axis can be downregulated through the receptor homodimer interfaces. Bacterial superantigens elicit a cytokine storm by binding through a conserved domain [12] directly into the CD28 [13] and B7 dimer interfaces [14], enhancing thereby the interaction of cell-surface CD28 with B7-2 as well as with B7-1 coligand [14, 15]. By contrast, we show that short peptide mimetics derived from distinct regions within the composite, self-adhesive CD28 dimer interface bind into the CD28 dimer interface and thereby inhibit B7-2/CD28 as well as B7-1/CD28 engagement underlying formation of intercellular B7/CD28 synapses, to attenuate signaling through CD28 for inflammatory cytokine expression. Moreover, we created short peptide mimetics of the B7-1 and B7-2 dimer interfaces and show that they exhibit tight selectivity for the cognate receptor in their ability to inhibit costimulatory axis formation between CD28 and its B7 co-receptors, yet each of the B7 mimetic peptides attenuates pro-inflammatory signaling through CD28. We exemplify the in-vivo relevance of this control mechanism in a prominent case of inflammatory cytokine storm, toxic shock induced by a bacterial superantigen [12]. B7-1 as well as CD28 homodimer interface mimetic peptides protect mice from lethal superantigen challenge even when dosed in molar amounts far below that of the superantigen. The explanation of this finding is that at submolar doses, the peptides protect not by competing with cell-surface B7 or CD28 receptors in binding to the superantigen, to prevent thereby engagement of the CD28 and B7 homodimer interfaces by the superantigen shown to be critical for its action [13, 14], but by attenuating signaling through CD28 via an inhibition of B7/CD28 costimulatory axis formation. These results show that the B7 and CD28 homodimer interfaces each control B7/CD28 receptor engagement and signaling through the B7/CD28 axis and demonstrate the broad protective potential of attenuating, via their remote dimer interfaces, B7/CD28 receptor engagement, to prevent thereby harmful overexpression of inflammatory cytokines.

## Materials and methods

### Peptides

Peptides were synthesized using fluoronyl-methoxycarbonyl chemistry, cleaved and the side chain deprotected with triflouroacetic acid. Peptides were abutted with D-Ala at both termini for greater protease resistance in biological assays. To allow coupling to the BIAcore chip for surface plasmon resonance, peptides were abutted with Cys at both termini [13, 14]. Peptides were > 95% pure by high-pressure liquid chromatography; molecular weight was verified by MALDI-TOF mass spectrometry.

### Antibodies

Mouse monoclonal anti-CD28 (MAB342, clone 37407) and anti-CD3 (clone UCHT1) [14], goat polyclonal anti-CD28 and anti-B7-2 (R&D Systems), and horseradish peroxidase-conjugated goat anti-mouse IgG or donkey anti-goat (KPL) antibodies were used.

### Induction of cytokine expression

Human PBMC from individual healthy donors were separated on Ficoll Paque (Amersham), washed three times with 50 ml of RPMI 1640 medium, resuspended at 4 × 10^6^ cells/ml and cultured in this medium supplemented with 2% fetal calf serum, 2 mM glutamine, 10 mM MEM nonspecific amino acids, 100 mM Na-pyruvate, 10 mM Hepes pH 7.2, 100 U/ml penicillin, 100 µg/ml streptomycin and 5 µg/ml nystatin (Biological industries) [13]. For each experiment examining induction of cytokines, PBMC prepared freshly from a single donor were used. Induction was done with 0.1 µg/ml αCD3, 2.5 µg/ml αCD28, or both. Highly purified recombinant SEB [13] was added to 100 ng/ml. Secreted cytokines were quantitated with Quantikine ELISA kits (R&D Systems).

### Soluble B7-2 and CD28

Recombinant human B7-2 (CD86) Fc chimera and human CD28 Fc chimera expressed in mouse myeloma NS0 cells (R&D Systems) comprise the extracellular 20–239 and 19–152 amino acid domain, respectively, of the mature human ligands fused to C-terminal human IgG1 Fc and are homodimers, disulfide-linked in the Fc domain. Soluble ligands were > 95% pure as judged by SDS-PAGE. To express monomeric CD28 extracellular domain protein without Fc, CD28 cDNA [14] served as template for PCR, using primers 5′-GGGAATCCAATGAACAAGATTTTGGTGAAG and 5′-GGACTGCAGTTATTAGGGCTTAGAAGGTCCGGG. The product was cloned into pHTT7K [16] and expressed in *E. coli* Rosetta(DE3)pLysS (Invitrogen) as N-terminally hexahistidine-tagged protein*.*

### Surface plasmon resonance spectroscopy

Peptides having Cys at both termini instead of D-Ala were diluted to 10–20 µg/ml in 10 mM Na acetate pH 4.0 and immobilized on a CM5 sensorchip using amine-thiol coupling kit (BIAcore). Analytes were injected at 20 μl/min in 25 mM HEPES pH 7.4, 150 mM NaCl, 3.4 mM EDTA, and 0.005% surfactant P20 under low ligand density conditions that minimize mass transfer limitations; a maximal binding capacity of the immobilized ligand in the range of 50–150 response units enables measurement of binding kinetics in the linear ligand concentration range (1:1 Langmuir binding). Regeneration was with 50 mM phosphoric acid. Kinetic analyses were performed at 25 °C in a BIAcore 3000 instrument, deducting the control flow cell signal from the binding signal. Analyte curves were run in duplicate; representative results are shown. BIAevaluation 3.1 software was used to determine dissociation constant KD in the linear ligand concentration range (1:1 Langmuir binding) [13, 14]. Human IgG (Jackson Laboratories) and ribonuclease A (Sigma) served as controls.

### CD28 and B7 expression vectors

Vectors expressing cell-surface CD28, CD28 fused C-terminally to GFP, cell-surface B7-2 and B7-2 or B7-2C fused C-terminally to Cherry have been described [13, 14]. Vector expressing B7-1 was generated by cDNA synthesis of human CD80 (NM_005191.3) from total human PBMC RNA using Verso RT-PCR kit (ABgene). CD80 cDNA was generated using KOD polymerase (Novagen) with phosphorylated PCR primers 5′-GACGTCGACATGGGCCACACACGGAGG and 5′-CACGCGGCCGCTTATACAGGGCGTACACTTTCCC. The PCR product was inserted into pEGFP-N3 DNA (Clontech) that had been digested with SalII and NotI and lacked the GFP region, using Fast-Link DNA Ligation Kit (Epicentre). Vector expressing B7-1 fused C-terminally to Cherry was generated from B7-1 cDNA vector template with phosphorylated PCR primers 5′-TACTCGAGATGGGCCACACACGGAGG and 5′-GTCCGCGGTACAGGGCGTACACTTTCCCTTC, deleting the *B7-1* termination codon. Upon digestion with XhoI and SacII, the PCR product was inserted into pmCherry-N1 DNA (Clontech).

### B7/CD28 interaction

To assay the effect of peptides on binding of B7-2 to CD28 on the cell, HEK-293T cell cultures were transiently transfected to express cell-surface CD28 or with empty vector expressing GFP with > 75% efficiency using Turbofect Transfection Reagent (Thermo Scientific) and 6 µg of expression vector DNA per 5 ml of cells at a density of 10^5^/ml. After 36 h, the cells were incubated for 45 min with 0.2 µg/ml soluble B7-2 in the absence or presence of peptide. After three washes with cold phosphate-buffered saline, cells were lysed. Equal amounts of total cell protein (Bradford assay) were subjected to 10% (wt/vol) PAGE and western blotting to show binding of B7-2 and expression of CD28 by the cells. Conversely, the effect of peptides on binding of CD28 to B7-2 on the cell was assayed by transfecting HEK-293T cells to express cell-surface B7-2. After 36 h, the cells were incubated for 45 min with 0.2 µg/ml soluble CD28 in the absence or presence of peptide. After three washes as above, cells were lysed. Equal amounts of total cell protein were subjected to 10% (wt/vol) PAGE and western blotting to show binding of CD28 and expression of B7-2 by the cells.

To assay the effect of peptides on intercellular B7-2/CD28 engagement by flow cytometry, vectors expressing CD28/GFP and B7-2/mCherry fusion proteins were used that leave the extracellular ligand binding domains intact. HEK-293T cells, separately transfected to express CD28/GFP (green) and B7-2/mCherry or B7-2C/mCherry (red), were co-incubated for 3 h in 24-well plates at room temperature at a concentration of 10^5^ cells/ml each. Cells were fixed using 1% formaldehyde in PBS at room temperature for 15 min and then washed with staining buffer (1% bovine serum albumin in phosphate-buffered saline). Receptor engagement between cell populations was analyzed by flow cytometry (Eclipse Flow Cytometry System, Sony), scoring the percentage of events positive for green and red using FlowJo vX.0.6 software [14], normalized to the transfection efficiency. Contour plots were generated using FlowJo vX.0.6 software. Receptor engagement between cells expressing CD28/GFP and B7-1/mCherry was assayed likewise.

### Ethics approval

Experiments involving superantigen challenge of mice were approved by the Institutional Animal Care and Use Committee (IACUC) of The Hebrew University-Hadassah Medical School. IACUC approval was for *n* = 10 mice/group. However, in the course of these experiments, the Animal Facility had a site visit by the Israel Ministry of Health who then limited experiments involving lethality to *n* = 5 mice/group.

### Lethal toxic shock

Female BALB/c mice (10 to 12 wk; Harlan) were challenged by intraperitoneal injection of 7.5 µg SEB and 20 mg of d-galactosamine to sensitize the animals to superantigens [12]. Antagonist peptides in phosphate-buffered saline or phosphate-buffered saline alone were injected intraperitoneally 30 min before SEB challenge. Survival was monitored over multiple time points. Viability remained constant for as long as monitored (7 d). Survival curves were analyzed using the Kaplan–Meier method, with the Gehan–Breslow–Wilcoxon test for comparisons.

### Structure modeling

Protein structures were modeled in PyMol (www.pymol.org) and Chimera (www.cgl.ucsf.edu/chimera/).

## Results

### CD28 dimer interface mimetic peptide attenuates intercellular signaling through CD28

**Fig. 1:**
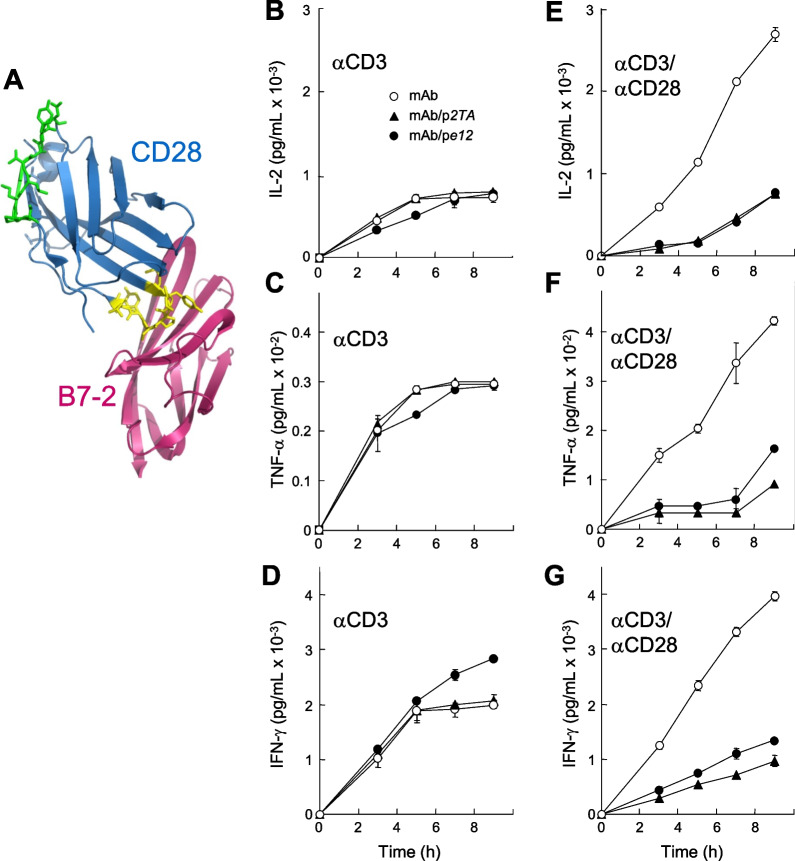
CD28 dimer interface mimetic peptide p*2TA* inhibits signaling through CD28. **A** The complex between CD28 (blue) and B7-2 (pink). The extracellular domain of CD28 is oriented such that it enters the T cell at the top and that of B7-2 is oriented such that it enters the antigen-presenting cell at the bottom. In CD28, p*2TA* sequence within the dimer interface is shown in green and the B7 binding site (MYPPPY) in yellow. Because the structure of the CD28/B7-2 complex remains unresolved, CD28 (1YJD.pdb [7]) was superimposed on CTLA-4 in the CTLA-4/B7-2 complex (1I85.pdb [14]). **B**–**G** PBMC from a single human donor were induced with αCD3 (**B**–**D**) or αCD3/αCD28 monoclonal antibodies (mAb) (**E**–**G**) alone (○) or in the presence of 10 µg/ml of p*2TA* (▲) or p*e12* (●). At times shown, IL-2, TNF-α and IFN-γ in culture medium were quantitated in triplicate. Data are mean and SEM. Representative data of 3 experiments are shown

In the extracellular domain of CD28, the homodimer interface and the binding site for B7 coligand are located at opposite poles (Fig. 1A). To examine the potential role of the CD28 homodimer interface in CD28 signaling, we investigated whether octapeptide p*2TA* (SPMLVAYD), a mimetic of residues 8–15 in the CD28 dimer interface [13] (Fig. 1A), might inhibit signaling through CD28. As positive control, we used p*e12* (SHFTHNRHGHST), a peptide selected by phage display for its affinity for the superantigen-binding site in CD28, which is the homodimer interface [13]. In human peripheral blood mononuclear cells (PBMC), neither p*2TA* nor p*e12* inhibited αCD3-mediated induction of interleukin-2 (IL-2), interferon-γ (IFN-γ) and tumor necrosis factor-α (TNF-α) (Fig. 1B–D), showing that they do not block signaling through the T cell receptor. However, each peptide strongly inhibited, yet did not ablate, induction of these inflammatory cytokines by αCD3 jointly with αCD28, a model for conventional T cell activation [13, 17] (Fig. 1E–G). As shown previously, *IL-2* and *IFN-γ* mRNA expression in human PBMC increase significantly upon induction by αCD3 together with αCD28 as compared to induction by αCD3 alone [13]. By itself, either peptide was devoid of IL-2, IFN-γ or TNF-α agonist activity (Additional file 1: Fig. S1). Thus, p*2TA* and p*e12* attenuate signaling for an inflammatory cytokine response when it is transduced through CD28.

### CD28 dimer interface mimetic peptide binds the CD28 dimer interface

**Fig. 2:**
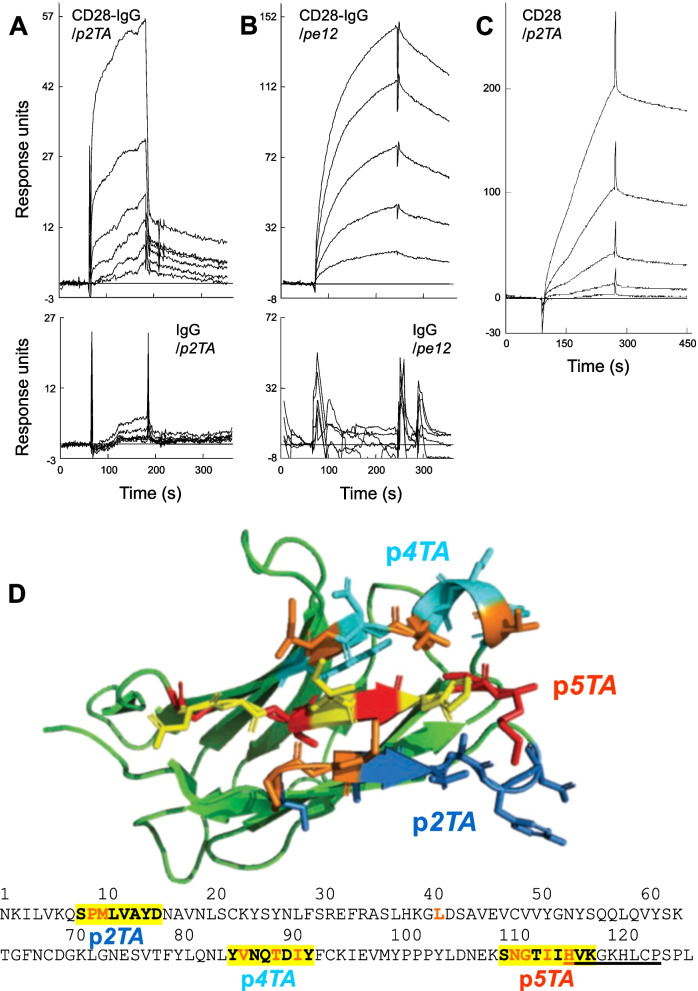
p*2TA* binds directly to CD28. **A**, **B** Representative surface plasmon resonance responses for binding of CD28-IgG-Fc to immobilized peptides p*2TA* (700 resonance units) (**A**) and p*e12* (1260 resonance units) (**B**) (top panels); K_D_, 4 and 3 µM, respectively. Analyte concentrations increased in twofold increments from 0.2 µM. Representative surface plasmon resonance responses for binding of IgG-Fc to immobilized p*2TA* and p*e12* are shown in bottom panels; analyte concentrations increased in twofold increments from 0.125 µM. **C** Representative surface plasmon resonance responses for binding of disulfide-linked homodimeric CD28 extracellular domain protein without Fc (CD28) to immobilized p*2TA* (733 resonance units). Analyte concentrations increased in twofold increments from 0.125 µM; K_D_, 3.4 µM. **D** The p*2TA*, p*5TA* and p*4TA* regions at the homodimer interface of CD28. In the sequence of the extracellular domain of CD28, dimer interface contact residues are shown in red color, peptide sequences p*2TA*, p*4TA* and p*5TA* are highlighted in yellow, and the αCD28 epitope [13] is underlined. In structure model of the extracellular domain of costimulatory receptor CD28 (green; 1yjd.pdb), a single beta-barrel, region of p*2TA* is shown in sticks in dark blue with 2 dimer interface contacts in orange, region of p*5TA* is in red with 4 dimer interface contacts in yellow and on the right the HVK sequence shared with the epitope, and region of p*4TA* is in cyan with 3 dimer interface contacts in orange

Considering that p*e12* was selected for its affinity for the CD28 dimer interface whereas p*2TA* is derived from the CD28 dimer interface that is inherently self-adhesive, we next examined whether p*2TA* can bind CD28. Indeed, p*2TA* and p*e12* each bound directly to soluble monomeric CD28, comprised of its extracellular domain fused to IgG1-Fc dimer (CD28-Fc) (Fig. 2A, B). Binding was background-subtracted and measured within the linear ligand concentration range. This binding was specific for CD28, as both peptides lacked affinity for IgG-Fc. Moreover, p*2TA* bound free CD28, provided as the recombinant extracellular domain protein without Fc that will form disulfide-linked homodimers in the non-reducing watery environment (Fig. 2C). Since p*e12* engages the dimer interface of CD28, this strengthens the concept that p*2TA* also binds there. Indeed, p*2TA* inhibits cytokine induction in human PBMC mediated by αCD28 (Fig. 1E–G), a monoclonal antibody whose epitope maps into the CD28 dimer interface at a sequence that although located over one hundred residues downstream of the p*2TA* domain [13], is in close proximity to it within the folded CD28 protein molecule [7] (Fig. 2D). Like p*e12*, p*2TA* hinders the effective action of αCD28 (Fig. 1E–G), further supporting the concept that p*2TA* binds back into the CD28 dimer interface. Notably, binding of p*2TA* to its CD28 target occurs with moderate, low micromolar affinity (Fig. 2A, C).

### CD28 dimer interface controls intercellular B7/CD28 engagement

**Fig. 3:**
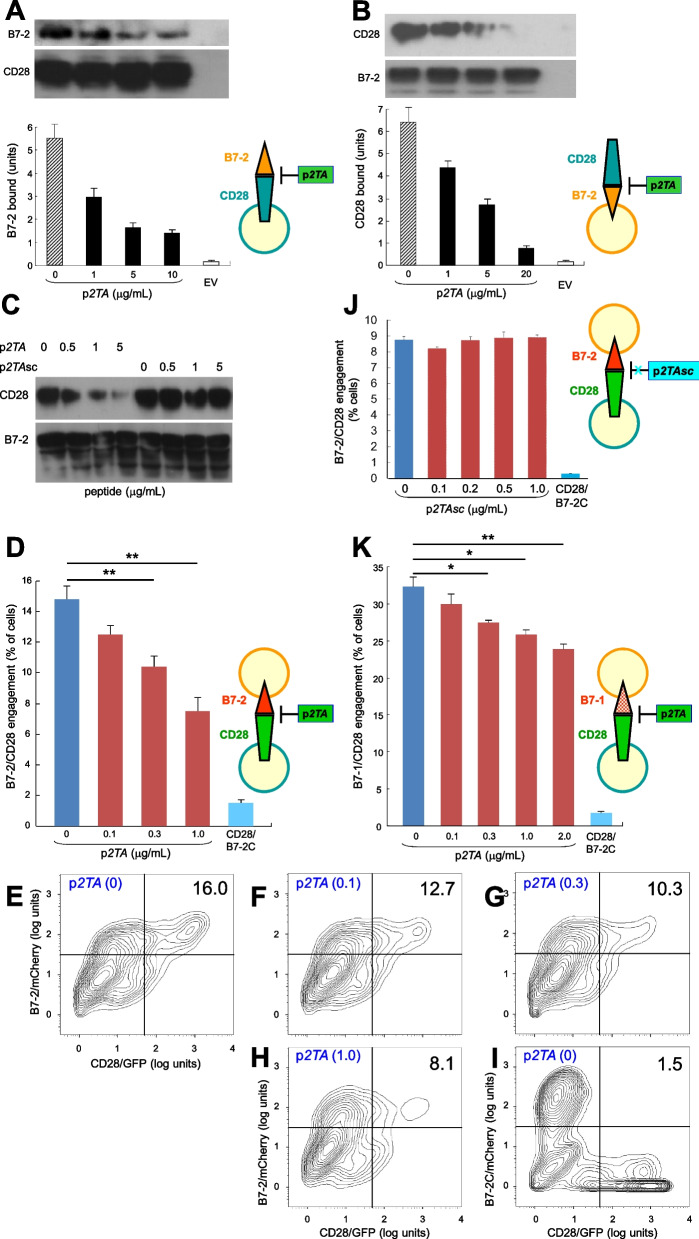
CD28 dimer interface mimetic peptide p*2TA* inhibits intercellular engagement of costimulatory receptor CD28 with B7-2 or B7-1. **A** p*2TA* inhibits binding of B7-2 to cell-surface CD28. HEK293T cells were transfected to express cell-surface CD28 or with empty vector (EV). Cells were incubated with soluble B7-2 in the absence or presence of p*2TA* at concentrations shown. Western blots show binding of B7-2 and equal expression of CD28 by the cells. Bound B7-2 is quantitated in the bar graphs; data are mean and SEM of three independent experiments. **B** p*2TA* inhibits binding of CD28 to cell-surface B7-2. HEK293T cells were transfected to express cell-surface B7-2 or with empty vector. Cells were incubated with soluble CD28 in the absence or presence of p*2TA* at concentrations shown. Western blots show binding of CD28 and equal expression of B7-2 by the cells. Bound CD28 is quantitated in the bar graphs; data are mean and SEM of three independent experiments. **C** HEK293T cells were transfected to express cell-surface B7-2. Cells were incubated with soluble CD28 in the absence or presence of p*2TA* or its randomly scrambled form, p*2TAsc* [13], at concentrations shown. Western blots show binding of CD28 and expression of B7-2 by the cells. **D** p*2TA* attenuates intercellular B7-2/CD28 receptor engagement. HEK293T cells transfected to express CD28/GFP fusion protein (green label) were incubated with HEK293T cells transfected to express B7-2/mCherry fusion protein (red label), in absence or presence of p*2TA* at concentrations shown. As negative control served B7-2C/mCherry, which lacks the ability to bind CD28. Intercellular B7-2/CD28 receptor engagement was scored using flow cytometry to quantitate per cent doubly labeled cells. Data are mean and SEM of three independent experiments. **E**–**I** Contour plots for a representative experiment in **D**, upon incubation of cells expressing CD28/GFP with cells expressing B7-2/mCherry (**E**–**H**) or B7-2C/mCherry (**I**) in the absence or presence of p*2TA* (µg/mL). Per cent doubly labeled cells is denoted in upper right quadrant. **J** p*2TAsc* fails to attenuate intercellular B7-2/CD28 engagement, assayed as in **D**. Data are mean and SEM of three independent experiments (contour plots: Additional file 1: Fig. S2). **K** p*2TA* attenuates intercellular B7-1/CD28 engagement. Synapse formation was assayed as in **D**, using B7-1/mCherry fusion protein instead of B7-2/mCherry (contour plots: Additional file 1: Fig. S3). Intercellular receptor engagement was compared using the one-tailed unpaired student’s t-test; *p < 0.05, **p < 0.005

In the folded extracellular domain of CD28, the homodimer interface is remote from the site where coligands B7-1 and B7-2 bind [7–10] (Fig. 1A), yet we hypothesized that the binding of p*2TA* and p*e12* to the dimer interface of CD28 might induce conformational change affecting its ability to engage B7. To examine this concept, we expressed CD28 or B7-2 on the cell surface and studied the effect of p*2TA* on binding of soluble B7-2 and CD28, respectively. This strategy allowed for monitoring the B7-2/CD28 interaction in the absence of multiple ligand-receptor interactions that underlie synapse formation between antigen-presenting cells and T cells, involving not only major histocompatibility class II/T-cell receptor interaction but also numerous costimulatory ligand pairs that could mask the contribution of B7-2/CD28 engagement [14]. Use of HEK293T cells avoids interactions between antigen-presenting cells and T cells, putting the focus on the B7-2/CD28 interaction. p*2TA* inhibited binding of B7-2 to cell-surface CD28 (Fig. 3A). No binding was seen with empty vector, hence it is CD28-dependent. Conversely, p*2TA* inhibited binding of CD28 to cell-surface B7-2 by over an order of magnitude (Fig. 3B). This inhibitory activity was dose-dependent. Random scrambling of the p*2TA* sequence, to ASMDYPVL, abrogated the ability of p*2TA* to inhibit binding of CD28 to cell-surface B7-2, showing that it is sequence-specific (Fig. 3C).

We next used two-color flow cytometry to validate that p*2TA* attenuates B7-2/CD28 engagement occurring specifically between the two cell populations that express B7-2 and CD28, respectively, in their native state on the cell membrane (Fig. 3D; representative contour plots in Fig. 3E–I). B7-2C, a splice variant of B7-2 unable to bind CD28 [18], failed to support significant intercellular receptor engagement, demonstrating specificity. Flow cytometry will not distinguish a receptor interaction formed by a few intercellular B7-2/CD28 pairs from one supported by numerous pairs, rendering it less sensitive than binding of the soluble costimulatory ligands (Fig. 3A, B). Yet, despite its moderate affinity for CD28 (Fig. 2A–C), p*2TA* had a pronounced and dose-dependent inhibitory effect on intercellular B7-2/CD28 engagement (Fig. 3D). Random scrambling of p*2TA* sequence sufficed to abrogate this property (Fig. 3J and Additional file 1: Fig. S2).

Moreover, p*2TA* progressively attenuated intercellular B7-1/CD28 engagement, which was not only more extensive but also more resistant to inhibition by p*2TA* than B7-2/CD28 engagement (Fig. 3D vs. K and Additional file 1: Fig. S3), reflecting the significantly higher affinity of B7-1 for CD28 [19].

Phage display peptide p*e12* likewise attenuated intercellular engagement between CD28 and B7-2 (Additional file 1: Fig. S4A) as well as that between CD28 and B7-1 (Additional file 1: Fig. S4B). Representative contour plots for B7-2/CD28 engagement are shown in Additional file 1: Fig. S4C–H and for B7-1/CD28 engagement formation in Additional file 1: Fig. S4I–M.

These results provide a mechanism for how p*e12* and CD28 dimer interface mimetic peptide p*2TA* attenuate CD28 signaling (Fig. 1E–G) by binding CD28 (Fig. 2A–C): via the CD28 dimer interface, they inhibit B7/CD28 engagement, downregulating formation of the primary costimulatory axis critical for T-cell activation. The interaction between CD28 and its two B7 coligands thus can be attenuated through the CD28 dimer interface.

### CD28 dimer interface peptides regulate CD28 signaling and engagement of B7 coligands

To examine whether the CD28 dimer interface sequence from which p*2TA* is derived has unique properties, we created p*4TA*, an octapeptide derived from a part of the homodimer interface located 78 amino acids downstream within the extracellular domain sequence of CD28 and p*5TA*, a nonapeptide derived from a part of the homodimer interface located more than one hundred amino acids downstream (Fig. 2D). Within the folded CD28 molecule, the p*2TA*, p*4TA* and p*5TA* regions lie adjacent, creating the homodimer interface (Fig. 2D). Whereas p*2TA* contains two amino acids that make dimer interface contacts, p*4TA* contains three dimer interface contact residues and p*5TA* four (Fig. 2D).

**Fig. 4:**
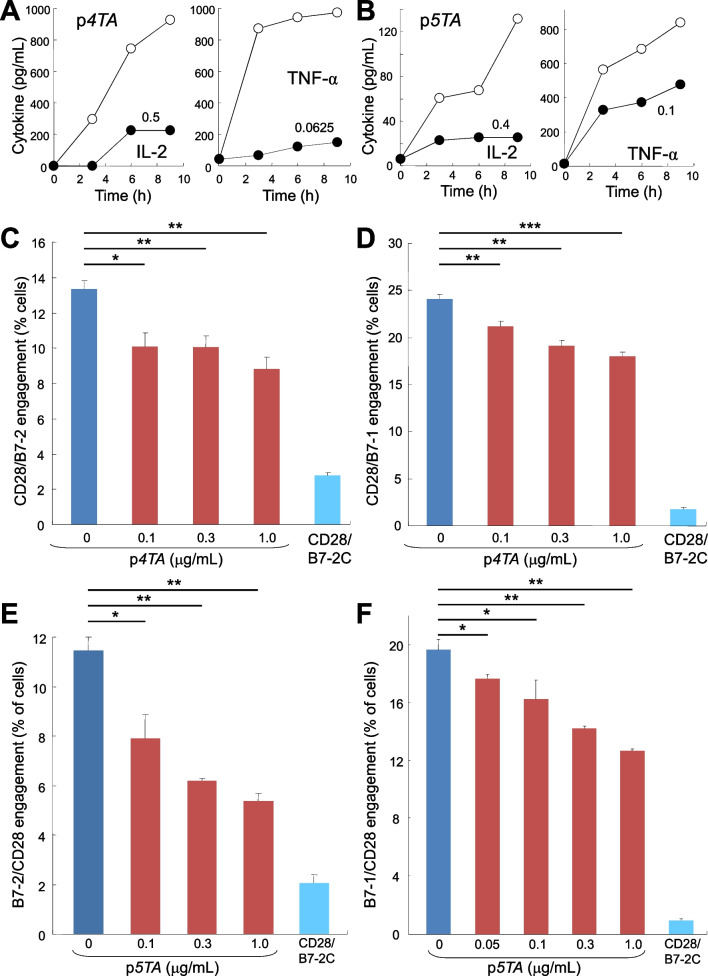
CD28 dimer interface mimetic peptides p*4TA* and p*5TA* attenuate B7/CD28 engagement, signaling through CD28, and inflammatory cytokine production. **A**, **B** Human PBMC were induced with αCD3/αCD28 monoclonal antibodies alone (open symbols) or in the presence of p*4TA* (**A**) or p*5TA* (**B**) at concentrations shown (µg/ml) (filled symbols). At times shown, IL-2 and TNF-α in culture medium were quantitated. Data in **A** and **B** are for PBMC from distinct human donors. Representative data of 3 experiments are shown. **C**, **D** p*4TA* attenuates intercellular B7/CD28 engagement. Receptor engagement was assayed by flow cytometry as in Fig. 3D for B7-2/CD28 engagement and as in Fig. 3K for B7-1/CD28 engagement. Data are mean and SEM of three independent experiments (contour plots: Additional file 1: Fig. S5A and S5B). **E**, **F** p*5TA* attenuates intercellular B7/CD28 engagement. Receptor engagement was assayed by flow cytometry as in **C** and **D**. Data are mean and SEM of three independent experiments (contour plots: Additional file 1: Fig. S5C, D). Intercellular receptor engagement was compared using the one-tailed unpaired student’s t-test; *p < 0.05, **p < 0.005, ***p < 0.001

Low concentrations of p*4TA* attenuated, yet did not ablate, signaling through CD28 for induction of IL-2 and TNF-α in human PBMC by αCD3/αCD28 (Fig. 4A). CD28 mimetic peptide p*5TA* likewise was capable of attenuating expression of IL-2 and TNF-α in human PBMC induced by αCD3/αCD28 (Fig. 4B). The range of cytokine expression induced, especially of IL-2, can vary for PBMC from individual donors, yet attenuation by the peptides is consistently observed.

As seen in Fig. 4A, B that use PBMC from distinct donors, the extent of attenuation by peptide mimetics was equal for IL-2 levels of 1000 pg/ml (Fig. 4A) and 130 pg/ml Fig. 4B induced by αCD3/αCD28. Thus, attenuation by peptide mimetics is not context-dependent and is detectable regardless of the extent of stimulation.

Flow cytometry analysis showed that p*4TA* attenuates intercellular B7-2/CD28 receptor engagement (Fig. 4C and Additional file 1: Fig. S5A) as well as intercellular B7-1/CD28 receptor engagement (Fig. 4D and Additional file 1: Fig. S5B). As for p*4TA*, low concentrations of p*5TA* progressively inhibited intercellular engagement between B7-2 and CD28 as well as between B7-1 and CD28 (Fig. 4E, F, Additional file 1: Fig. S5C and S5D). p*5TA* progressively attenuated intercellular B7-1/CD28 receptor engagement, which was not only more extensive but also more resistant to inhibition by p*5TA* than B7-2/CD28 engagement (Fig. 4E vs. F), reflecting the significantly higher affinity of B7-1 for CD28 [19].

Like p*2TA* and p*e12*, p*4TA* and p*5TA* impeded the effective action of αCD28 (Fig. 4A, B), supporting the concept that these peptide mimetics bind back into the CD28 dimer interface. The action of p*4TA* and of p*5TA* significantly broadens the scope of CD28 dimer interface mimetic peptides as regulators of B7/CD28 receptor engagement and demonstrates a role for the entire CD28 dimer interface in pro-inflammatory signaling.

### B7-1 and B7-2 dimer interface mimetic peptides attenuate engagement of CD28 by the cognate B7 receptor

**Fig. 5:**
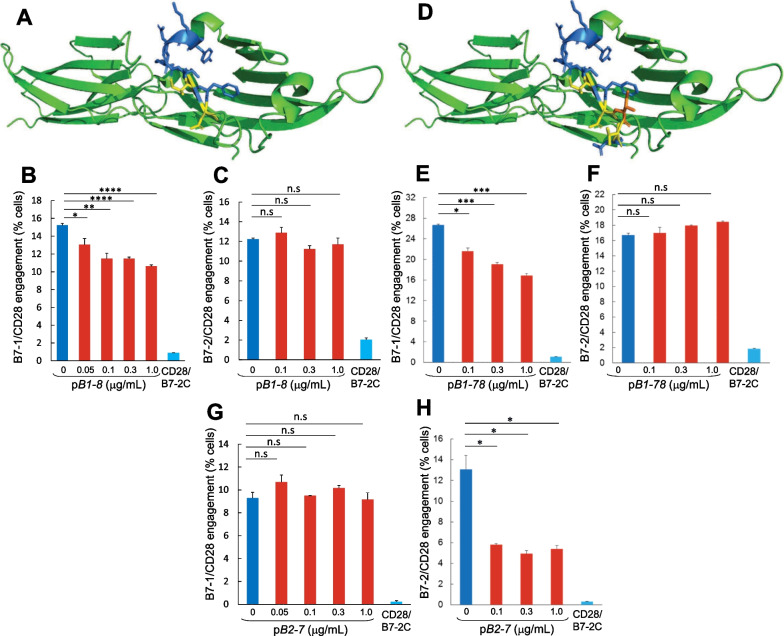
B7-1 and B7-2 dimer interface mimetic peptides attenuate engagement of CD28 by the cognate B7 costimulatory receptor. **A** In cartoon model of the extracellular domain of costimulatory receptor B7-1 (CD80) (green; 1dr9.pdb), a double beta-barrel, amino acid residues forming p*B1-8* are modeled in sticks, with 2 residues making homodimer interface contacts shown in yellow. **B**, **C** p*B1-8* selectively attenuates intercellular B7-1/CD28 engagement (**B**) but not B7-2/CD28 engagement (**C**). Receptor engagement was assayed by flow cytometry as in Fig. 3K for B7-1/CD28 engagement and as in Fig. 3D for B7-2/CD28 engagement. **D** In the extracellular domain of B7-1, amino acid residues forming p*B1-78* are modeled in sticks, with 4 residues making homodimer interface contacts shown in yellow and orange. **E**, **F** p*B1-78* selectively attenuates intercellular B7-1/CD28 engagement (**E**) but not B7-2/CD28 engagement (**F**). **G**, **H** p*B2-7* selectively attenuates intercellular B7-2/CD28 engagement (**H**) but not B7-1/CD28 engagement (**G**). Data are mean and SEM of three independent experiments (contour plots: Additional file 1: Fig. S6). Intercellular receptor engagement was compared using the one-tailed unpaired student’s t-test; *p < 0.05, **p < 0.005, ***p < 0.001, ****p < 0.0001; n.s, not significant

In the folded extracellular domain of B7-1, a homodimer (1DR9.pdb [8]), the dimer interface is remote from the site where B7-1 engages CTLA-4 in the human B7-1/CTLA-4 costimulatory complex (1I8L.pdb [10]), and by homology modeling, from the site where B7-1 engages CD28 [7]. We created B7-1 octapeptide p*B1-8* (YKNRTIFD), which carries two residues that make homodimer interface contacts (underlined) (Fig. 5A). Flow cytometry analysis showed that p*B1-8* attenuates intercellular B7-1/CD28 receptor engagement (Fig. 5B and Additional file 1: Fig. S6A) yet fails to inhibit intercellular B7-2/CD28 receptor engagement which occurs with far lower affinity [19] (Fig. 5C and Additional file 1: Fig. S6B). We also created B7-1 undecapeptide p*B1-78* (YKNRTIFDITN) with four residues that make homodimer interface contacts (Fig. 5D). Flow cytometry analysis showed that p*B1-78* attenuates intercellular B7-1/CD28 engagement (Fig. 5E and Additional file 1: Fig. S6C), yet as for p*B1-8*, fails to inhibit the weaker intercellular B7-2/CD28 engagement (Fig. 5F and Additional file 1: Fig. S6D).

We next examined the ability of a decapeptide mimetic of the weak B7-2 receptor homodimer interface, p*B2-7* (MGRTSFDSDS) containing nine residues that make homodimer interface contacts [14], to attenuate intercellular B7/CD28 engagement. In contrast to the B7-1 dimer interface mimetic peptides, p*B2-7* failed to attenuate intercellular B7-1/CD28 engagement (Fig. 5G and Additional file 1: Fig. S6E) yet this peptide effectively attenuated intercellular B7-2/CD28 engagement (Fig. 5H and Additional file 1: Fig. S6F).

Thus, whereas CD28 homodimer interface mimetic peptides are capable of attenuating intercellular engagement of both B7-1 and B7-2 coligands by CD28 (Figs. 3 and 4), B7 homodimer interface mimetic peptides exhibit tight selectivity for the cognate receptor in their ability to inhibit intercellular engagement between cell-surface CD28 and its B7 co-receptors (Fig. 5A–H). This selectivity reinforces the concept that the costimulatory receptor dimer interface mimetic peptides bind back into the self-adhesive dimer interface they are derived from, and thereby regulate the ligand interactions of the cognate receptor.

**Fig. 6:**
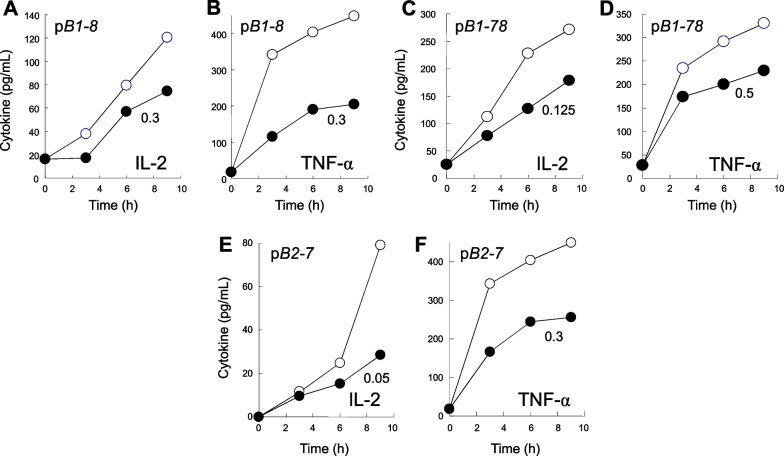
B7-1 and B7-2 dimer interface mimetic peptides attenuate signaling through CD28 and inflammatory cytokine production. **A**–**F** Human PBMC were induced with αCD3/αCD28 monoclonal antibodies alone (open symbols) or in the presence of p*B1-8* (**A**, **B**), p*B1-78* (**C**, **D**) or p*B2-7* (**E**, **F**) at concentrations shown (µg/mL) (filled symbols). At times shown, IL-2 and TNF-α in culture medium were quantitated. PBMC from three distinct human donors were used to generate the data in **A**–**F**, respectively. Representative data of 3 experiments are shown

The ability of CD28 dimer interface mimetic peptides to inhibit signaling through CD28 by αCD3/αCD28 monoclonal antibodies (Figs. 1E–G, 4A, B) in principle could be accounted for by their ability to attenuate formation of the B7/CD28 costimulatory axis shown in Figs. 3 and 4, by interference with the binding of αCD28 into its epitope which overlaps, as mentioned, the CD28 dimer interface by 3 amino acids (Fig. 2D), or both. The cognate receptor specificity exhibited by the B7 dimer interface mimetic peptides, demonstrated above, provided a tool to analyze this question. Indeed, p*B1-8*, p*B1-78* as well as p*B2-7* each were capable of attenuating the induction of IL-2 and TNF-α when it was induced by exposure to αCD3/αCD28 (Fig. 6A–F). This finding strongly supports the conclusion that it is the ability of the B7 mimetic peptides to attenuate formation of the B7/CD28 costimulatory axis that underlies their ability to counteract pro-inflammatory signaling through CD28 for cytokine production. Although αCD3/αCD28 activate T cells independently from B7, the observed attenuation supports the conclusion that once they are activated, the T cells subsequently activate an inflammatory response that is B7-dependent and hence sensitive to the B7 mimetics.

### The dimer interface mimetic peptides protect mice from lethal toxic shock in doses far submolar to the superantigen

To induce an inflammatory cytokine storm, Gram-positive bacterial superantigens depend strictly on a conserved 12-amino-acid β-strand-hinge-α-helix domain, remote from their binding sites for T cell receptor and major histocompatibility complex class II molecules [12]. By binding via this domain into the homodimer interfaces of CD28 [13] and of B7-2 [14], superantigens strongly enhance intercellular costimulatory axis formation between B7-2 and CD28 [14]. Likewise, diverse superantigens strongly enhance intercellular costimulatory synapse formation between B7-1 and CD28 [15]. When present at sufficiently high concentrations, CD28 dimer interface mimetic peptide p*2TA* and B7-2 dimer interface mimetic peptide p*B2-7* compete with the cell-surface receptors in binding to the superantigen, inhibiting thereby access of the superantigen to its CD28 and B7-2 targets and preventing lethal toxic shock [13, 14]. We therefore predicted that as was shown for p*2TA* and p*B2-7* [13, 14], mimetic peptides p*4TA* and p*5TA*, as well as p*B1-8* and p*B1-78*, should attenuate the induction of IL-2 and TNF-α in human PBMC by the major superantigen, staphylococcal enterotoxin B (SEB). As seen from Additional file 1: Fig. S7, this was indeed the case.

**Fig. 7:**
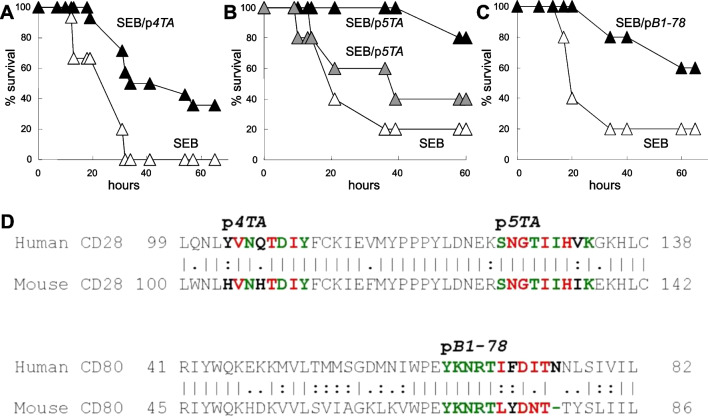
At doses submolar to toxin, CD28 and B7-1 dimer interface mimetic peptides protect mice from lethal superantigen challenge. **A** Mice were injected with SEB alone (*n* = 15 per group) (open symbols) or together with 0.04 µg p*4TA* (*n* = 14 per group) (black symbols); p for survival, 0.062. **B** Mice (*n* = 5 per group) were injected with SEB alone or together with 0.045 µg p*5TA* (grey symbols) or 0.09 µg p*5TA* (black symbols); p for survival, 0.029. **C** Mice (*n* = 5 per group) were injected with SEB alone or together with 0.052 µg p*B1-78* (black symbols); p for survival, 0.192. **D** Protein sequences of human and mouse CD28 and CD80, aligned by EMBOSS Needle. Sequences shown are portions of human (P10747) and mouse (P31041) CD28 and of human (P33681) and mouse (Q3U4B5) CD80. Dimer interface contacts in p*4TA*, p*5TA* and p*B1-78* are shown in red, non-contact residues are shown in green if identical and in black if non-identical

Given that the induction of an inflammatory cytokine response in human PBMC, whether caused by αCD3/αCD28 or by SEB, was inhibited by p*4TA* and p*5TA* that attenuate formation of the B7/CD28 costimulatory axis (Fig. 4 and Additional file 1: Fig. S7), we considered that these CD28 dimer interface mimetic peptides might be capable of protecting mice from lethal superantigen challenge even when dosed in molar amounts well below that of the superantigen. Indeed, SEB induced pronounced mortality within hours, yet when a low dose of p*4TA* or p*5TA* was administered at the time of exposure to SEB, each of these peptides was able to provide protection from death (Fig. 7A, B). Mice that received either p*4TA* (Fig. 7A) or p*5TA* (Fig. 7B, grey symbols) at a dose sevenfold lower than that of SEB in terms of molar amount, showed marked survival benefit. Mice that received p*5TA* at a dose 3.5-fold lower than that of SEB in terms of molar amount, showed near-complete survival (Fig. 7B, black symbols). Likewise, mice that received p*B1-78* at a dose sevenfold submolar to SEB showed marked survival (Fig. 7C).

The target of the superantigen and of the dimer interface mimetic peptides is identical: the homodimer interface of the cognate CD28/B7 receptor [13–15] (Figs. 2A–C, 3D–K, 4C–F, 5). Hence, cellular targets of SEB and of peptide mimetics are expressed in vivo at the very same level. SEB kills mice within hours (Fig. 7A–C) and the peptides protect within the same time range. Protection by peptide is dose-dependent (Fig. 7B). These observations indicate that the bioavailability and pharmacokinetics of SEB and of the peptide mimetics are similar.

All dimer interface contact residues within the p*4TA*, p*5TA* and p*B1-78* sequences are conserved between human and mouse CD28 and CD80, respectively, except for two out of four in p*B1-78* (one mismatch I:L, one I·N) (Fig. 7D)*.*

The CD28 and B7-1 dimer interface mimetic peptides thus protect effectively from lethal superantigen challenge even when dosed in molar amounts far below that of the toxin. The explanation of this finding is that at submolar doses, the peptides protect not by competing with CD28 and B7-1 for the superantigen but by attenuating signaling through CD28 via an inhibition of B7/CD28 costimulatory axis formation.

## Discussion

These results reveal a novel, fundamental mechanism that regulates the inflammatory response to microbial pathogens through control of B7/CD28 costimulatory receptor engagement. Formation of the B7/CD28 costimulatory axis is controlled through the B7 and CD28 receptor homodimer interfaces. We demonstrate the protective potential against cytokine storm of attenuating pro-inflammatory signaling via these protein domains. Selectively targeting formation of the B7/CD28 costimulatory axis through the receptor homodimer interfaces provides a mechanism for attenuating lethal host inflammatory responses to infections, exemplified here with superantigen-induced toxic shock. Within the B7-1(CD80), B7-2(CD86) and CD28 extracellular domains, the dimer interface is located far from the coligand-binding site (Fig. 1A) [7–10], yet we show that B7-2/CD28 as well as B7-1/CD28 engagement is controlled through the remote dimer interfaces. We studied the action of short peptide mimetics derived from the B7-1 and B7-2 dimer interface and from distinct regions within the CD28 dimer interface. Binding of a dimer interface mimetic peptide into its cognate dimer interface most plausibly induces allosteric change in the receptor that acts to diminish its affinity for coligand, thus down-regulating costimulatory synapse formation and intercellular signal transduction through the B7/CD28 axis, reducing thereby inflammatory cytokine induction. This underscores the regulatory role of the homodimer interfaces in pro-inflammatory signaling and renders them therapeutic targets against systemic inflammatory responses. Structures of the CD28/B7 complexes were not resolved, yet our results support the conclusion that within each of the CD28 and B7 molecules, interaction between the homodimer interface domains is necessary for productive coligand engagement. The comparable ability of peptides derived from regions in the self-adhesive CD28 dimer interface that are widely separated across the linear CD28 sequence yet in close proximity within the folded receptor protein, to inhibit the low-affinity B7-2/CD28 costimulatory interaction as well as the far tighter B7-1/CD28 engagement and to attenuate intercellular signal transduction through CD28 for inflammatory cytokine expression, reveals a broad regulatory role for the CD28 homodimer interface in B7 coligand engagement during formation of the immunological synapse between T cell and antigen-presenting cell. We demonstrate attenuation of IL-2 induction, a cytokine specific for T cells. That shows that the peptide mimetics attenuate proinflammatory signalling downstream of CD28.

Attenuation of cytokine induction in human PBMC by the homodimer interface mimetics occurs promptly, within 3–6 h, rendering it unlikely that they affect the surface expression levels of CD28 and B7-1/2. We show that the peptides attenuate, yet do not ablate, cytokine induction. Hence, CD28 remains functional. It is highly unlikely that the short homodimer interface mimetic peptide by itself would significantly alter half-life or internalization of CD28.

CD28 dimer interface mimetic peptide p*2TA* engages CD28 with micromolar affinity (Fig. 2A, C), i.e., a moderate affinity. The interaction of CD28 with its two B7 coligands also occurs with micromolar affinity [19]. By contrast, CTLA-4 binds B7-2 as well as B7-1 an order of magnitude more tightly than does CD28 [19]. For this reason, CTLA-4/Ig [20] (abatacept) deprives not only CD28 of its B7 coligands but also cell-surface CTLA-4, thereby counteracting the function of CTLA-4 in limiting the inflammatory response and disturbing the homeostatic balance between pro- and anti-inflammatory signaling, causing side effects [21, 22]. By attenuating, yet not eliminating, inflammatory signaling through a moderate affinity for B7 and CD28, the mimetic peptides provide a more selective approach that while preventing cytokine storm, leaves a basal response intact, to allow a return to immune homeostasis with no compromise of host defenses, essential to enable elimination of the pathogen.

The concept that long-range allosteric effects within CD28 can control its function is supported by the findings that mutation of K118/K120 in the αCD28 epitope used here, located at the CD28 dimer interface, with K118 overlapping the *C*-terminal residue of mimetic peptide p*5TA* (Fig. 2D), can enhance the avidity of CD28 for B7-1 [23] and that by binding into the CD28 [13] and B7 dimer interface [14] through a conserved 12-amino-acid β-strand-hinge-α-helix domain remote from their major histocompatibility class II and T cell receptor binding sites [12], bacterial superantigens enhance intercellular synapse formation mediated by the interaction of cell-surface CD28 with B7-2 as well as with B7-1 coligand [14, 15], eliciting a hyperinflammatory response. By binding directly to the superantigen, short peptide mimetics of the CD28 or B7-2 dimer interface compete with the cell-surface receptors for the superantigen and thereby inhibit access of the superantigen to these receptors [13, 14, 24]. Thus, when administered in sufficiently high doses, such peptides protect from lethal superantigen-induced toxic shock [13, 14, 24]. In the present study, however, dimer interface mimetic peptides protected mice from lethal superantigen challenge even when dosed in molar amounts far below that of the superantigen (Fig. 7), precluding competition with cell-surface CD28 and B7 for superantigen as the underlying mechanism. The plausible explanation of this finding is that the peptides protect by attenuating signaling through CD28 via an inhibition of its engagement with B7 coreceptors.

Not only does binding of a superantigen into the CD28 or B7 homodimer interface elicit a cytokine storm by triggering the interaction between CD28 and its B7 coligands [14, 15], but an αCD28 monoclonal antibody whose epitope maps close to the dimer interface induces vigorous inflammatory cytokine expression in human PBMC (Fig. 1). As shown here, by contrast, short peptide mimetics of the costimulatory receptor homodimer interface engage their cognate B7 or CD28 dimer interface to induce thereby the exact opposite, attenuating the B7/CD28 interaction and diminishing the inflammatory response, an outcome that could not have been predicted from either superantigen or αCD28 mode of action.

Notably, CD28 dimer interface mimetic peptide p*2TA* protects mice not only from lethal infection with Gram-positive bacteria that are capable of producing superantigens [25] but even in the absence of superantigens, from lethal shock induced by lipopolysaccharide, a hallmark of Gram-negative bacteria, as well as from Gram-negative bacterial infection and from polymicrobial sepsis [26]. This strongly supports the concept that B7/CD28 signaling is broadly involved in the inflammatory pathogenesis of different infections. Currently there is no drug available against sepsis, yet this peptide completed US Phase 2 and Phase 3 human clinical trials against necrotizing soft tissue infections, a major form of severe sepsis; in a single dose, the peptide significantly enhanced resolution of multi-organ dysfunction and attenuated cytokine storm [27, 28]. In total-body irradiated mice, p*2TA* prevents inflammatory and thrombotic reactions and protects against gastrointestinal injury [29]. The mechanism underlying the broad protective activity against hyper-inflammation, bacterial infections and sepsis, even in the absence of superantigens, was hitherto not resolved. Our present findings provide the explanation: by inhibiting the B7/CD28 receptor interaction, the peptide downregulates the inflammatory response.

## Conclusions

The B7/CD28 interaction is regulated through the receptor homodimer interfaces, rendering these receptor domains critical targets for controlling inflammatory cytokine expression. This provides a molecular basis for the ability of dimer interface mimetic peptides to protect not only from superantigen-induced toxic shock but also from a broader spectrum of pathogenic bacteria. By attenuating the human inflammatory response through the B7/CD28 costimulatory axis, such peptides should be effective against a broad spectrum of pathogens that evoke a cytokine storm in the host.

## Supplementary Information

**Additional file 1: Figure S1.** p*2TA* and p*e12* do not induce a significant Th1 cytokine response. A–C Human PBMC were cultured with 10 µg/mL of p2TA *o*r p*e12* alone. At times shown, IL-2, TNF-α and IFN-γ secreted into the culture medium were determined in triplicate. Representative data of 3 experiments are shown. **Figure S2.** Random scrambling of p*2TA* sequence abrogates its ability to inhibit intercellular B7-2/CD28 synapse formation. A–F Contour plots are shown for a representative experiment in Fig. 3J, upon incubation of cells expressing CD28/GFP with cells expressing B7-2/mCherry or B7-2C/mCherry in the absence or presence of p*2TAsc*. Per cent doubly labeled cells is denoted in upper right quadrant. **Figure S3.** p*2TA* attenuates intercellular B7-1/CD28 synapse formation. A-F Contour plots for a representative experiment in Fig. 3K, upon incubation of cells expressing CD28/GFP with cells expressing B7-1/mCherry or B7-2C/mCherry, in the absence or presence of p*2TA*. Per cent doubly labeled cells is denoted in upper right quadrant. **Figure S4.** p*e12* attenuates intercellular B7/CD28 engagement. A p*e12* attenuates intercellular B7-2/CD28 synapse formation. HEK293T cells transfected to express CD28/GFP fusion protein were incubated with HEK293T cells transfected to express B7-2/mCherry fusion protein, in absence or presence of p*e12* at concentrations shown. As negative control served B7-2C/mCherry. Intercellular B7-2/CD28 synapse formation was scored using flow cytometry to quantitate per cent doubly labeled cells. Data are mean and SEM of three independent experiments. Intercellular synapse formation was compared using the one-tailed unpaired student’s t-test; *p < 0.05, **p < 0.005, ***p < 0.001. B p*e12* attenuates intercellular B7-1/CD28 synapse formation. Synapse formation was assayed as in A, using B7-1/mCherry fusion protein instead of B7-2/mCherry. C–H Contour plots for a representative experiment in A, upon incubation of cells expressing CD28/GFP with cells expressing B7-2/mCherry or B7-2C/mCherry. Incubation was done in the absence or presence of p*e12*. Per cent doubly labeled cells is denoted in upper right quadrant. I–M Contour plots are shown likewise for a representative experiment in B, upon incubation of cells expressing CD28/GFP with cells expressing B7-1/mCherry or B7-2C/mCherry. **Figure S5.** CD28 dimer interface mimetic peptides p*4TA* and p*5TA* attenuate B7/CD28 engagement. A, B p*4TA* attenuates intercellular B7/CD28 synapse formation. Contour plots for a representative experiment in Fig. 4C and in Fig. 4D, upon incubation of cells expressing CD28/GFP with cells expressing, respectively, B7-2/mCherry and B7-1/mCherry or B7-2C/mCherry. Incubation was done in the absence or presence of p*4TA*. Per cent doubly labeled cells is denoted in upper right quadrant. C, D p*5TA* attenuates intercellular B7/CD28 synapse formation. Contour plots for a representative experiment in Fig. 4E and in Fig. 4F, upon incubation of cells expressing CD28/GFP with cells expressing, respectively, B7-2/mCherry and B7-1/mCherry or B7-2C/mCherry. Incubation was done in the absence or presence of p*5TA*. Per cent doubly labeled cells is denoted in upper right quadrant. **Figure S6.** B7-1 and B7-2 dimer interface mimetic peptides attenuate engagement of CD28 by the cognate B7 receptor. A, B p*B1-8* selectively attenuates intercellular B7-1/CD28 engagement but not B7-2/CD28 engagement . Contour plots for a representative experiment in Fig. 5B and in Fig. 5C, upon incubation of cells expressing CD28/GFP with cells expressing, respectively, B7-1/mCherry and B7-2/mCherry. Incubation was done in the absence or presence of p*B1-8*. Per cent doubly labeled cells is denoted in upper right quadrant. C, D p*B1-78* selectively attenuates intercellular B7-1/CD28 engagement but not B7-2/CD28 engagement. Contour plots for a representative experiment in Fig. 5E and in Fig. 5F , upon incubation of cells expressing CD28/GFP with cells expressing, respectively, B7-1/mCherry and B7-2/mCherry. Incubation was done in the absence or presence of p*B1-78*. Per cent doubly labeled cells is denoted in upper right quadrant. E, F p*B2-7* selectively attenuates intercellular B7-2/CD28 engagement but not B7-1/CD28 engagement. Contour plots for a representative experiment in Fig. 5G and in Fig. 5H , upon incubation of cells expressing CD28/GFP with cells expressing, respectively, B7-1/mCherry and B7-2/mCherry. Incubation was done in the absence or presence of p*B2-7*. Per cent doubly labeled cells is denoted in upper right quadrant. B7-2C/mCherry control panels are indicated. **Figure S7.** CD28 and B7-1 dimer interface mimetic peptides attenuate superantigen-mediated induction of inflammatory cytokines. A–D Human PBMC from a single donor were induced with SEB alone or in the presence of p*4TA*, p*5TA*, p*B1-8 * or p*B1-78 *at concentrations shown. At times shown, IL-2 and TNF-α in culture medium were quantitated. Representative data of 3 experiments are shown.

## Data Availability

Source data are provided with this paper. All other datasets generated and analyzed in the current study are available from the corresponding author upon reasonable request.
