# Supplementary material for: The homodimer interfaces of costimulatory receptors B7 and CD28 control their engagement and pro-inflammatory signaling
Source: J Biomed Sci. 2023 Jun 28;30:49. doi: 10.1186/s12929-023-00941-3 (PMC10308680; doi:10.1186/s12929-023-00941-3)
Supplement: Supplementary file 1 — Additional file 1: Figure S1. p2TA and pe12 do not induce a significant Th1 cytokine response. A–C Human PBMC were cultured with 10 µg/mL of p2TA or pe12 alone. At times shown, IL-2, TNF-α and IFN-γ secreted into the culture medium were determined in triplicate. Representative data of 3 experiments are shown. Figure S2. Random scrambling of p2TA sequence abrogates its ability to inhibit intercellular B7-2/CD28 synapse formation. A–F Contour plots are shown for a representative experiment in Fig. 3J, upon incubation of cells expressing CD28/GFP with cells expressing B7-2/mCherry or B7-2C/mCherry in the absence or presence of p2TAsc. Per cent doubly labeled cells is denoted in upper right quadrant. Figure S3. p2TA attenuates intercellular B7-1/CD28 synapse formation. A-F Contour plots for a representative experiment in Fig. 3K, upon incubation of cells expressing CD28/GFP with cells expressing B7-1/mCherry or B7-2C/mCherry, in the absence or presence of p2TA. Per cent doubly labeled cells is denoted in upper right quadrant. Figure S4. pe12 attenuates intercellular B7/CD28 engagement. A pe12 attenuates intercellular B7-2/CD28 synapse formation. HEK293T cells transfected to express CD28/GFP fusion protein were incubated with HEK293T cells transfected to express B7-2/mCherry fusion protein, in absence or presence of pe12 at concentrations shown. As negative control served B7-2C/mCherry. Intercellular B7-2/CD28 synapse formation was scored using flow cytometry to quantitate per cent doubly labeled cells. Data are mean and SEM of three independent experiments. Intercellular synapse formation was compared using the one-tailed unpaired student’s t-test; *p < 0.05, **p < 0.005, ***p < 0.001. B pe12 attenuates intercellular B7-1/CD28 synapse formation. Synapse formation was assayed as in A, using B7-1/mCherry fusion protein instead of B7-2/mCherry. C–H Contour plots for a representative experiment in A, upon incubation of cells expressing CD28/GFP with cells expressing B7-2/mC [file 12929_2023_941_MOESM1_ESM.pdf]

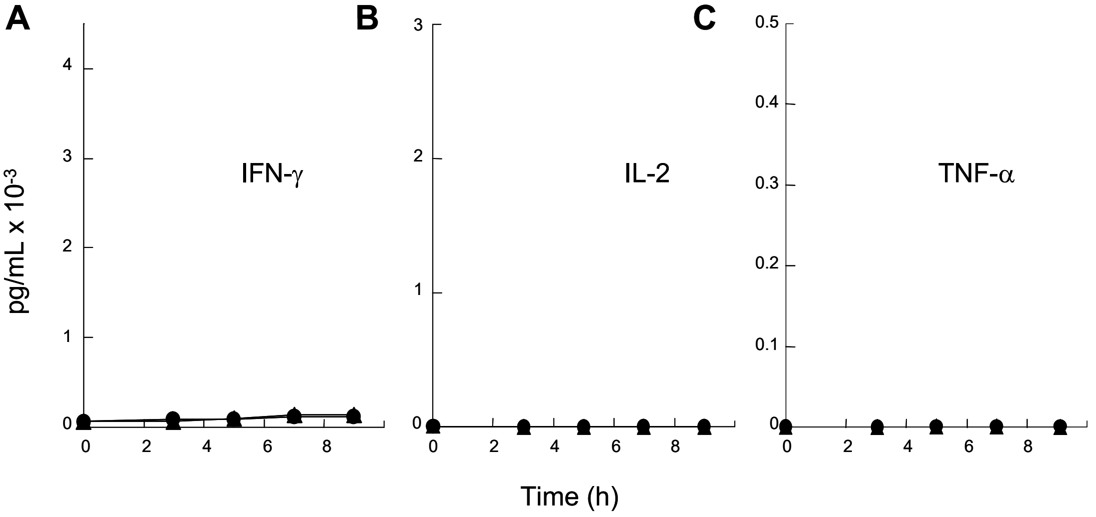

Fig. S1 p*2TA* and p*e12* do not induce a significant Th1 cytokine response. **A-C** Human PBMC were cultured with 10 μg/mL of p*2TA* (▲) or p*e12* (●) alone. At times shown, IL-2, TNF-α and IFN-γ secreted into the culture medium were determined in triplicate (error bars, SEM). Representative data of 3 experiments are shown.

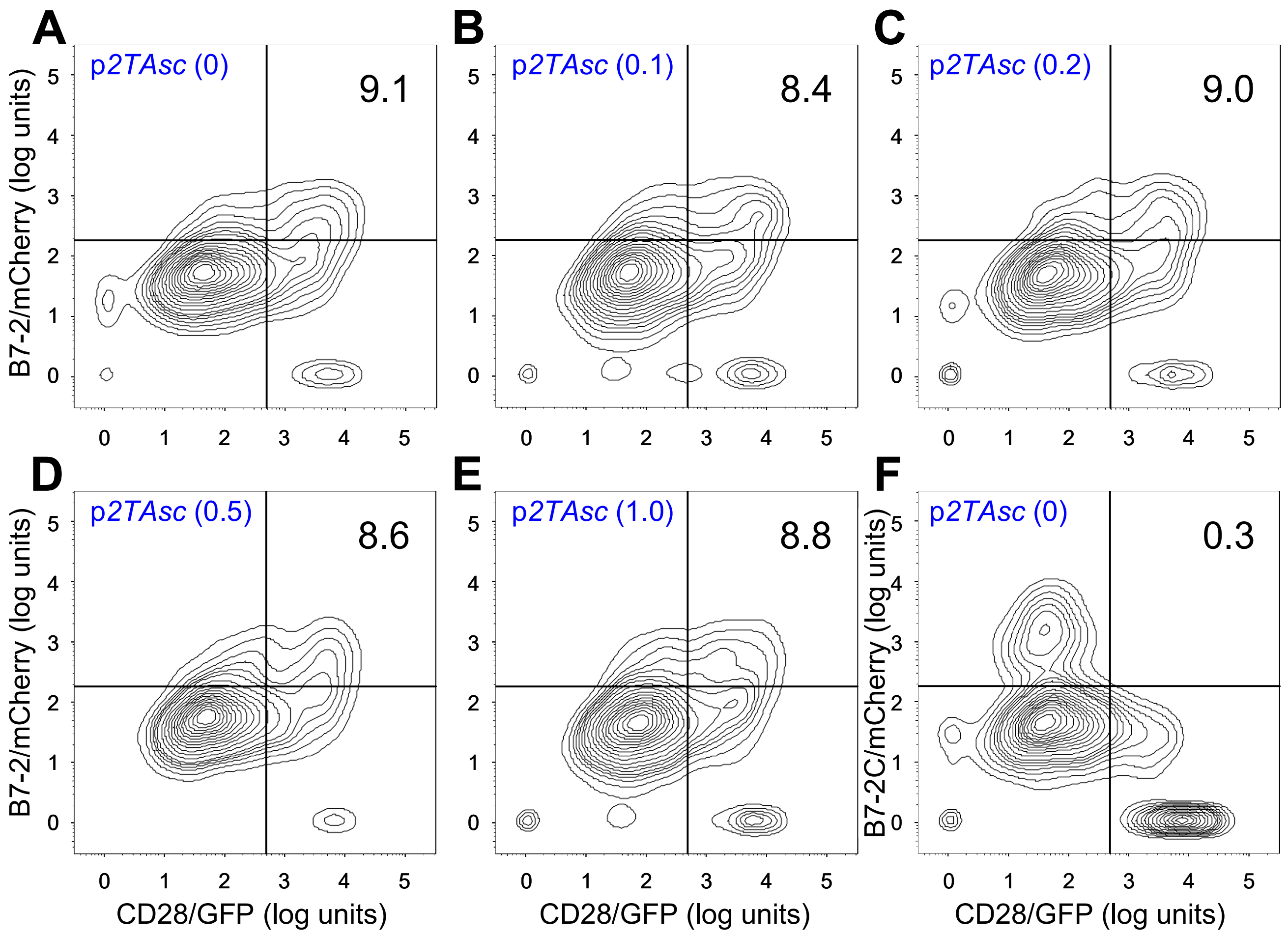

Fig. S2 Random scrambling of p*2TA* sequence abrogates its ability to inhibit intercellular B7-2/CD28 synapse formation. **A-F** Contour plots are shown for a representative experiment in Fig. 3J, upon incubation of cells expressing CD28/GFP with cells expressing B7-2/mCherry (**A-E**) or B7-2C/mCherry (**F**) in the absence or presence of p*2TAsc* (µg/mL). Per cent doubly labeled cells is denoted in upper right quadrant.

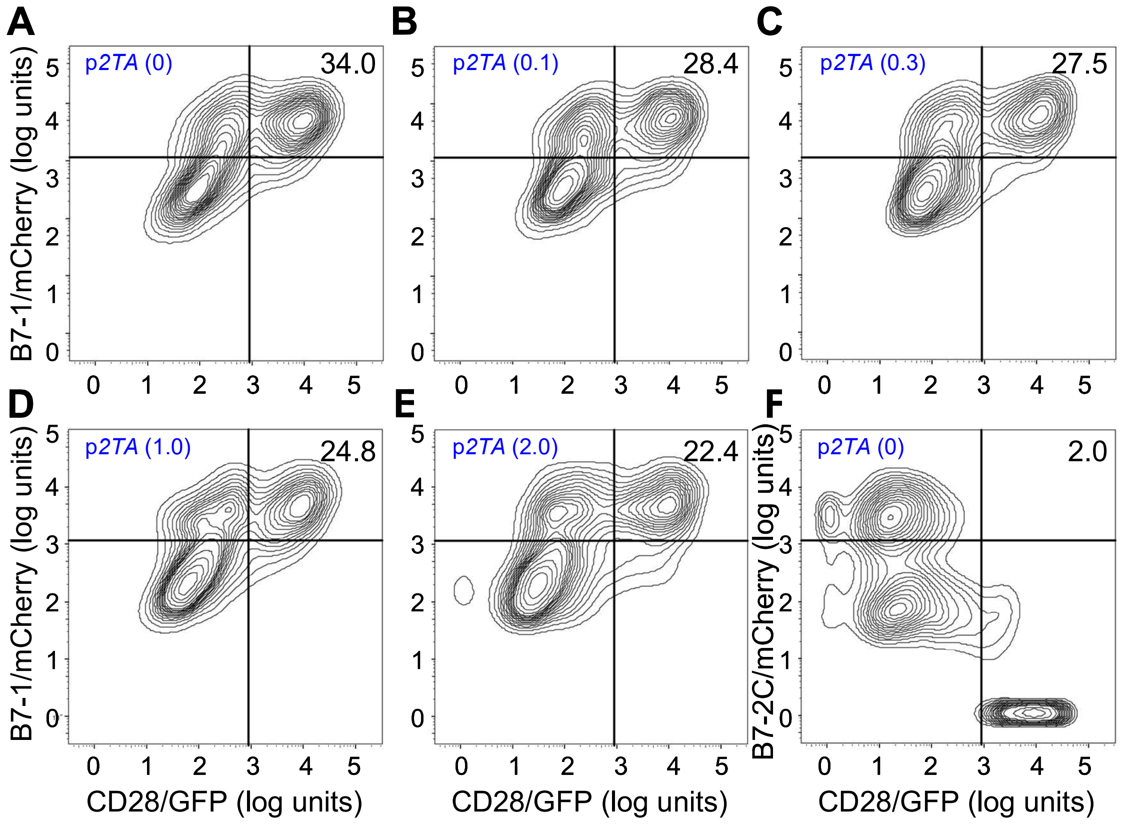

Fig. S3 p*2TA* attenuates intercellular B7-1/CD28 synapse formation. **A-F** Contour plots for a representative experiment in Fig. 3K, upon incubation of cells expressing CD28/GFP with cells expressing B7-1/mCherry (**A-E**) or B7-2C/mCherry (**F**), in the absence or presence of p*2TA* (μg/mL). Per cent doubly labeled cells is denoted in upper right quadrant.

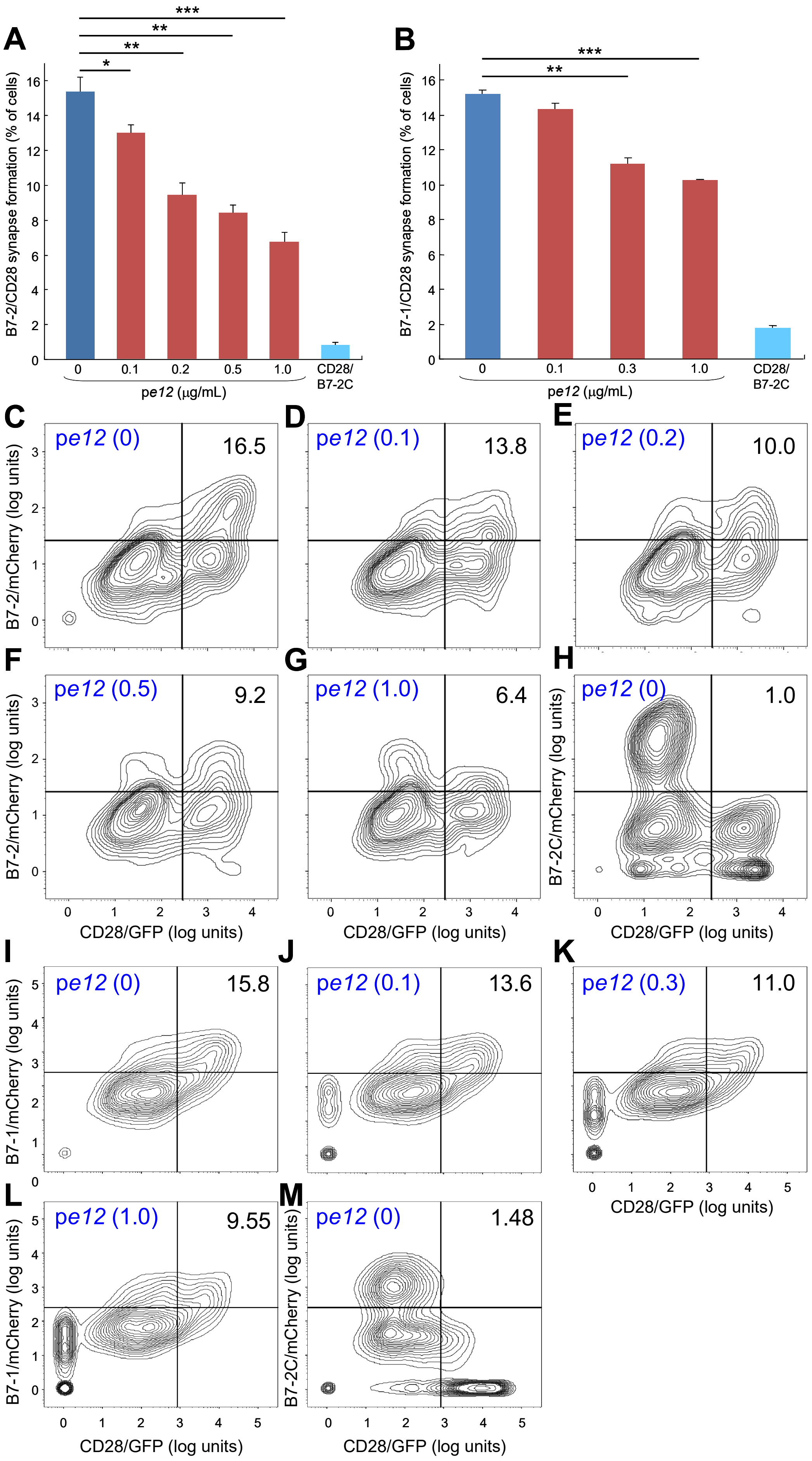

A
B7-2/CD28 synapse formation (% of cells)
***
**
**
*
0
0.1
0.2
0.5
1.0
CD28/ B7-2C
pe12 (μg/mL)
B
B7-1/CD28 synapse formation (% of cells)
***
**
0
0.1
0.3
1.0
CD28/ B7-2C
pe12 (μg/mL)
C
pe12 (0)
16.5
D
pe12 (0.1)
13.8
E
pe12 (0.2)
10.0
F
pe12 (0.5)
9.2
G
pe12 (1.0)
6.4
H
pe12 (0)
1.0
B7-2/mCherry (log units)
B7-2C/mCherry (log units)
CD28/GFP (log units)
I
pe12 (0)
15.8
J
pe12 (0.1)
13.6
K
pe12 (0.3)
11.0
L
pe12 (1.0)
9.55
M
pe12 (0)
1.48
B7-1/mCherry (log units)
B7-2C/mCherry (log units)
CD28/GFP (log units)

Fig. S4 p*e12* attenuates intercellular B7/CD28 engagement. **A** p*e12* attenuates intercellular B7-2/CD28 synapse formation. HEK293T cells transfected to express CD28/GFP fusion protein (green label) were incubated with HEK293T cells transfected to express B7-2/mCherry fusion protein (red label), in absence or presence of p*e12* at concentrations shown. As negative control served B7-2C/mCherry. Intercellular B7-2/CD28 synapse formation was scored using flow cytometry to quantitate per cent doubly labeled cells. Data are mean and SEM of three independent experiments. Intercellular synapse formation was compared using the one-tailed unpaired student's t-test; *p < 0.05, **p <0.005, ***p <0.001. **B** p*e12* attenuates intercellular B7-1/CD28 synapse formation. Synapse formation was assayed as in **A**, using B7-1/mCherry fusion protein instead of B7-2/mCherry. **C-H** Contour plots for a representative experiment in **A**, upon incubation of cells expressing CD28/GFP with cells expressing B7-2/mCherry (**C-G**) or B7-2C/mCherry (**H**). Incubation was done in the absence or presence of p*e12* (µg/mL). Per cent doubly labeled cells is denoted in upper right quadrant. **I-M** Contour plots are shown likewise for a representative experiment in **B**, upon incubation of cells expressing CD28/GFP with cells expressing B7-1/mCherry (**I-L**) or B7-2C/mCherry (**M**).

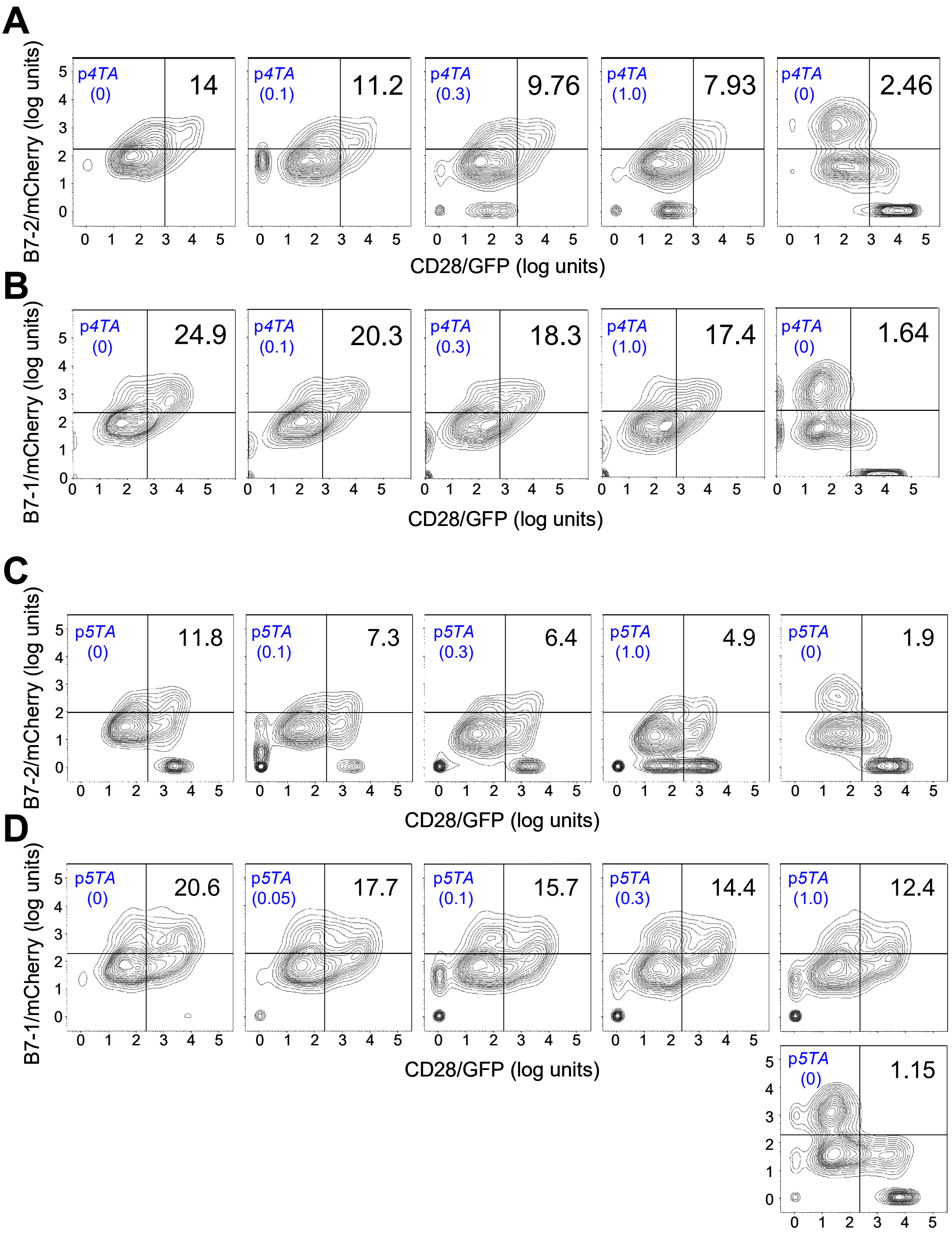

Fig. S5. CD28 dimer interface mimetic peptides p*4TA* and p*5TA* attenuate B7/CD28 engagement. **A, B** p*4TA* attenuates intercellular B7/CD28 synapse formation. Contour plots for a representative experiment in Fig. 4C (**A**) and in Fig. 4D (**B**), upon incubation of cells expressing CD28/GFP with cells expressing, respectively, B7-2/mCherry (**A**) and B7-1/mCherry (**B**) (4 panels from left) or B7-2C/mCherry (panel on right). Incubation was done in the absence or presence of p*4TA* (µg/mL). Per cent doubly labeled cells is denoted in upper right quadrant. **C, D** p*5TA* attenuates intercellular B7/CD28 synapse formation. Contour plots for a representative experiment in Fig. 4E (**C**) and in Fig.

4F (**D**), upon incubation of cells expressing CD28/GFP with cells expressing, respectively, B7-2/mCherry (**C**) (4 panels from left) and B7-1/mCherry (**D**) (5 upper panels) or B7-2C/mCherry (lower panel). Incubation was done in the absence or presence of p*5TA* (μg/mL). Per cent doubly labeled cells is denoted in upper right quadrant.

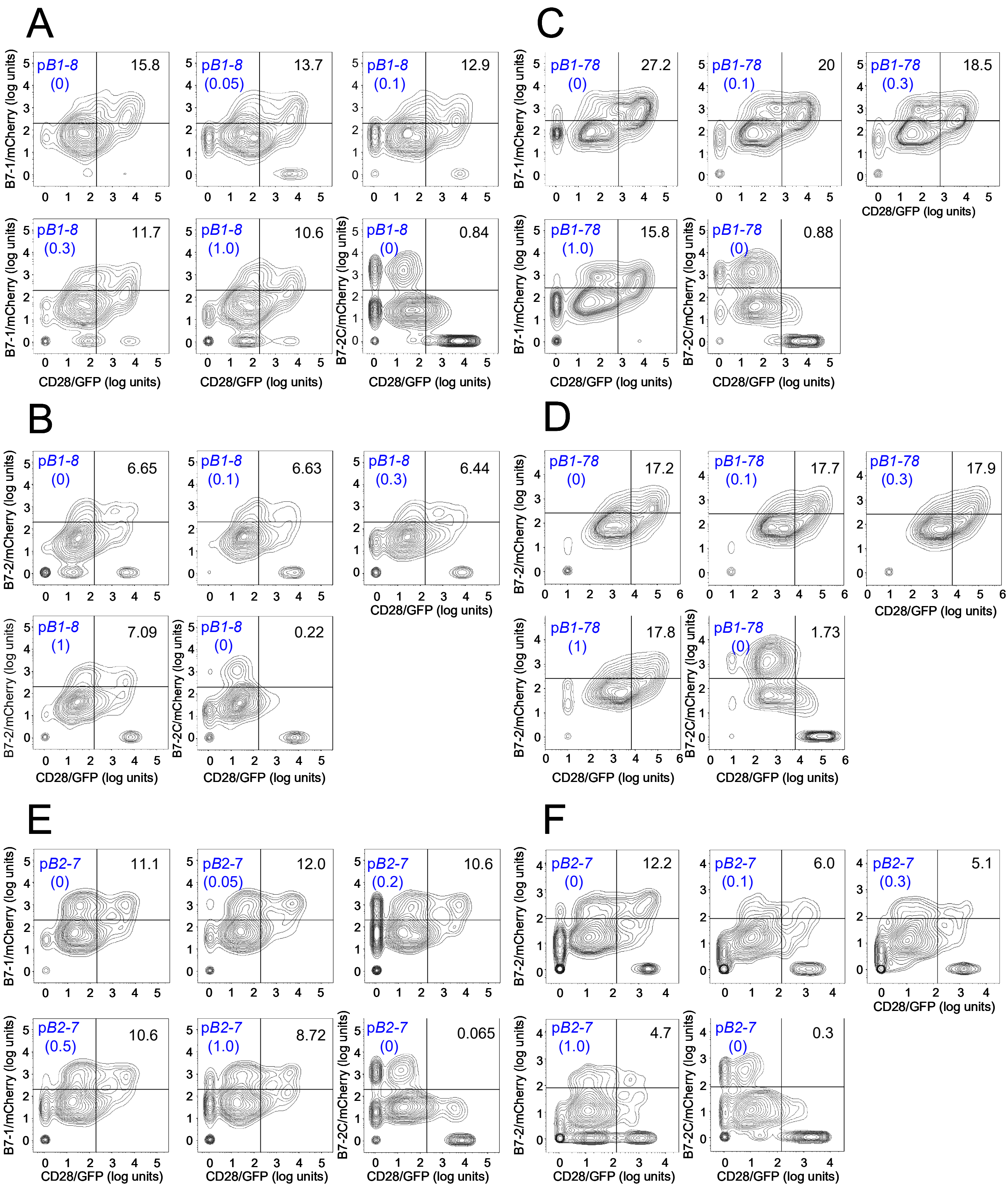

Supplementary Fig. S6 B7-1 and B7-2 dimer interface mimetic peptides attenuate engagement of CD28 by the cognate B7 receptor**. A, B** p*B1-8* selectively attenuates intercellular B7-1/CD28 engagement (**A**) but not B7-2/CD28 engagement (**B**). Contour plots for a representative experiment in Fig. 5B (**A**) and in Fig. 5C (**B**), upon incubation of cells expressing CD28/GFP with cells expressing, respectively, B7-1/mCherry (**A**) and B7-2/mCherry (**B**). Incubation was done in the absence or presence of p*B1-8* (µg/mL). Per cent doubly labeled cells is denoted in upper right quadrant. **C, D** p*B1-78* selectively attenuates intercellular B7-1/CD28 engagement (**C**) but not B7-2/CD28 engagement (**D**). Contour plots for a representative experiment in Fig. 5E (**C**) and in Fig. 5F (**D**), upon incubation of cells expressing CD28/GFP with cells expressing, respectively, B7-1/mCherry (**C**) and B7-2/mCherry (**D**). Incubation was done in the absence or presence of p*B1-78* (µg/mL). Per cent doubly labeled cells is denoted in upper right quadrant. **E, F** p*B2-7* selectively attenuates intercellular B7-2/CD28 engagement (**F**) but not B7-1/CD28 engagement (**E**). Contour plots for a representative experiment in Fig. 5G (**E**) and in Fig. 5H (**F**), upon incubation of cells expressing CD28/GFP with cells expressing, respectively, B7-1/mCherry (**E**) and B7-2/mCherry (**F**). Incubation was done in the absence or presence of p*B2-7* (µg/mL). Per cent doubly labeled cells is denoted in upper right quadrant. B7-2C/mCherry control panels are indicated.

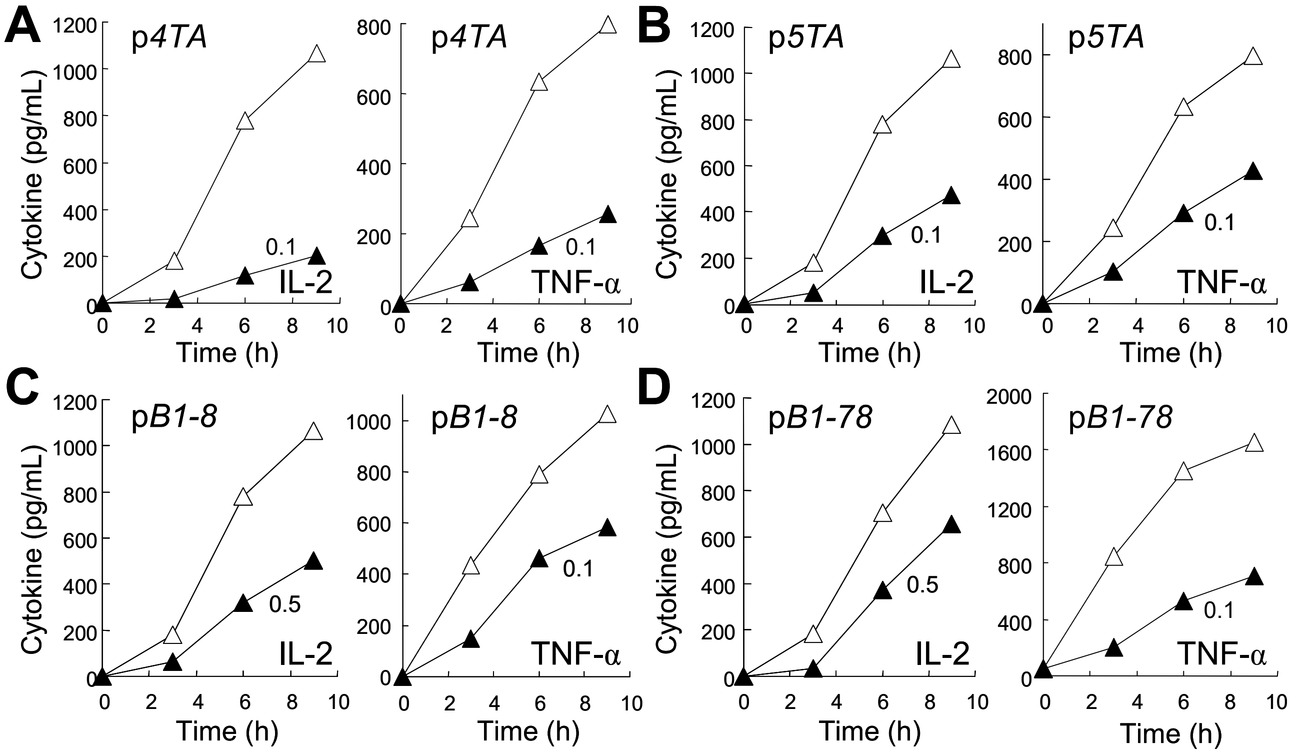

Fig. S7 CD28 and B7-1 dimer interface mimetic peptides attenuate superantigen-mediated induction of inflammatory cytokines. **A-D** Human PBMC from a single donor were induced with SEB alone (open symbols) or in the presence of p*4TA* (**A**), p*5TA* (**B**), p*B1-8* (**C**) or p*B1-78* (**D**) at concentrations shown (μg/mL) (filled symbols). At times shown, IL-2 and TNF-α in culture medium were quantitated. Representative data of 3 experiments are shown.
